# Correlation of simultaneous measurements of coronary, carotid and aortic distensibility and vessel wall ratio as evaluated by cardiovascular magnetic resonance in patients with type 2 diabetes mellitus

**DOI:** 10.1186/1532-429X-15-S1-P225

**Published:** 2013-01-30

**Authors:** David Jean Winkel, Tingting Xiong, Nikolaus Tiling, Rolf Gebker, Eckart Fleck, Ursula Plöckinger, Sebastian Kelle

**Affiliations:** 1 Internal Medicine/Cardiology, German Heart Institute Berlin, Berlin, Germany; 2 Interdisziplinäres Stoffwechsel-Centrum, Charité-Universitätsmedizin Berlin, Campus Virchow-Klinikum, Berlin, Germany

## Background

Arterial distensibility can be measured non-invasively by cardiovascular magnetic resonance (CMR) in various arterial territories. However, no information is available comparing distensibility in different anatomical territories in individual patients. Therefore, we sought to compare CMR measurements of the distensibility of the coronary and carotid artery, and ascending aorta (AA) in patients with type 2 diabetes mellitus (DM).

## Methods

A total of 31 patients (20 men, mean age 62 ± 10 years) with DM were studied using a commercial whole body MRI system at 3.0 Tesla (Philips, The Netherlands). In each patient, the proximal segment of a coronary artery, both common carotid arteries and a transversal scan at the level of the ascending aorta were imaged for cross-sectional area measurements using CMR. Distensibility (mmHg-1*103) was determined as (lumen max - lumen min)/(pulse pressure x lumen min) x 1000. For all vascular territories lumen area and total vessel area were assessed and vessel wall ratio (VWR) (vessel wall area/body surface area) was calculated. All continuous parameters are given as mean + one standard deviation (SD). For all tests, p<0.05 was considered statistically significant. All tests were two-sided.

## Results

Thirty patients had adequate image quality for common carotid artery, 29 patients for ascending aorta and 23 patients for coronary artery measurements. Distensibility of the common carotid artery, ascending aorta and coronary arteries was measured in patients with DM (3.0 ± 1.3 mmHg-1*103 vs. 3.6 ± 2.4 mmHg-1*103 and 2.4 ± 1.7 mmHg- 1*103 respectively). We found no significant correlation of carotid vs. aortic distensibility (R=0.122; p=0.528), carotid vs. coronary distensibility (R=0.234; p=0.295) or aortic vs. coronary distensibility (R=0.112; p=0.627). In addition, we found no significant correlation of distensibility vs. VWR in the common carotid artery (R=-0.072; p=0.720), ascending aorta (R=-0.027; p=0.902) or coronary artery (R=-419; p=0.228).

## Conclusions

In patients with type 2 diabetes we found no significant correlation between distensibility of different vascular territories or when comparing distensibility and VWR in each single vessel territory. These findings suggest that the assessment of early atherosclerotic vascular changes in peripheral vessels is limited for individual cardiovascular risk stratification in patients with type 2 diabetes.

## Funding

none

